# Radial neck fracture presenting to a Chiropractic clinic: a case report and literature review

**DOI:** 10.1186/2045-709X-22-14

**Published:** 2014-04-01

**Authors:** Clinton J Daniels, Jordan A Gliedt, Dennis E Enix

**Affiliations:** 1 Private Practice, St. Louis, MO, USA; 2 Private Practice, Chandler, AZ, USA; 3 Logan University, Chesterfield, MO, USA

**Keywords:** Elbow fracture, Radial head fracture, Chiropractic

## Abstract

**Objective:**

The purpose of this case report is to describe a patient that presented with a Mason type II radial neck fracture approximately three weeks following a traumatic injury.

**Clinical features:**

A 59-year old female presented to a chiropractic practice with complaints of left lateral elbow pain distal to the lateral epicondyle of the humerus and pain provocation with pronation, supination and weight bearing. The complaint originated three weeks prior following a fall on her left elbow while hiking.

**Intervention and outcome:**

Plain film radiographs of the left elbow and forearm revealed a transverse fracture of the radial neck with 2mm displacement--classified as a Mason Type II fracture. The patient was referred for medical follow-up with an orthopedist.

**Conclusion:**

This report discusses triage of an elbow fracture presenting to a chiropractic clinic. This case study demonstrates the thorough clinical examination, imaging and decision making that assisted in appropriate patient diagnosis and management.

## Background

Traumatic injuries to the forearm are a common occurrence in the emergency room setting. They occasionally present in the private practice setting, especially as a chronic presentation. It is easy for the clinician to ignore the obvious diagnosis of fracture due to the length of time the patient has endured this condition. Fractures of the radial head are relatively common. They represent approximately 5.4 percent of all fractures, between 1.5 and 4.5 percent of fractures in adults, and approximately one third of all fractures of the elbow [1-5]. The vast majority of these fractures occur in individuals between the ages of 30-60 years, with a mean age between 45 and 45.9 years and are more common in women than men [6]. It has been reported that in children the incidence of radial head and neck fractures is up to 1.3% [6]. Mechanism of injury is usually a fall on an outstretched arm, and in rare cases, direct trauma [1-3,6-9]. These fractures are typically seen in isolation, but may be accompanied by other fractures, dislocations or soft tissue injuries. Because of their proximity, the medial collateral ligament, lateral collateral ligament and interosseous ligaments are most prone to injury with radial head fractures. The radial head of the elbow acts as a secondary stabilizer of the joint creating 30% of the elbow’s resistance to valgus forces as such it is prone to compressive forces and hyperextension injuries.

Symptoms typically include pain and tenderness along the lateral aspect of elbow and a limited range of motion (ROM) in the elbow, forearm or wrist. A thorough physical exam should include an inspection of ROM and longitudinal and rotational joint stability, evaluating the joint for resistance to elbow flexion and extension and forearm pronation and supination; stressing varus and valgus positions of the joint. Particular attention should be paid to the presence of pain and tenderness in the interosseous membrane.

**Figure 1:**
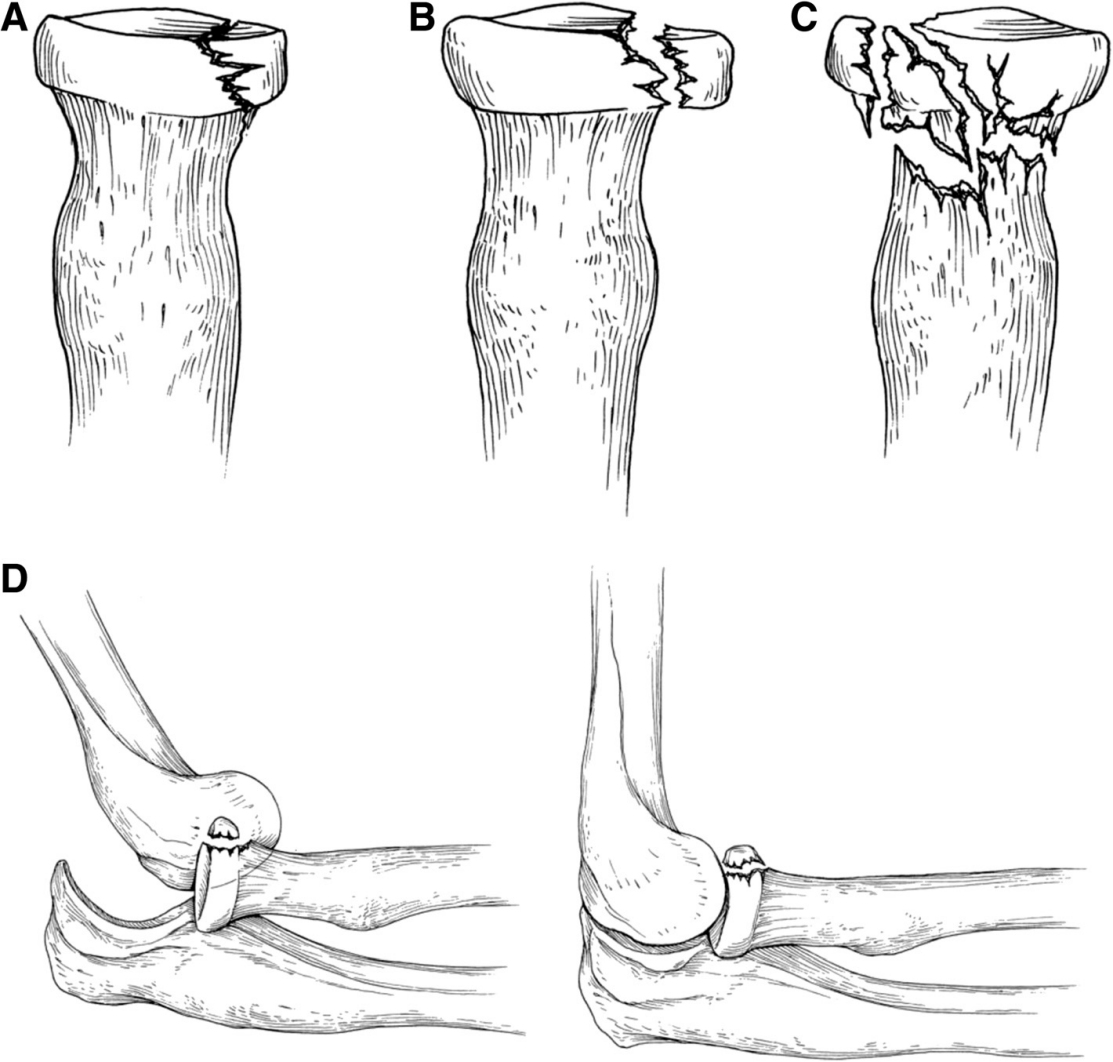
**Mason classification of radial head/neck fractures. A**, Type I, nondisplaced. **B**, Type II, displaced partial articular fracture of ≥2mm displacement or ≥30% of the articular surface. **C**, Type III, comminuted fracture. **D**, A type IV injury, described by Johnson [14] in 1962, indicates an associated ipsilateral ulnohumeral dislocation. Image from Terrible Triad Injury of the Elbow: Current Concepts, JAAOS March 2009 vol. 17 no. 3 137-151 [15].

Patients presenting with a mechanism of injury consistent with known fractures should be examined radiographically. Radial head fracture presentations are described by the Mason classification guideline. Mason, originally described three types: non-displaced fractures (Type I); displaced partial head fractures (Type II); and comminuted, displaced fractures involving the entire head (Type III) [10]. In an attempt to quantify the extent of radial head involvement, Broberg and Morrey proposed that a partial radial head fracture (Type II) must be of adequate size and movement to be considered displaced, suggesting fracture of at least 30 percent of the auricular surface and 2 mm of displacement [11,12]. Further modification was proposed by Johnston who sought to include fractures of the radial head with associated elbow dislocation (Type IV) [13]. This modified Mason classification system (Figure 1) is widely considered the principal radial head fracture subgrouping system, and is often referenced at time of diagnosis and treatment.

## Case presentation

### Clinical history

A 59 year-old female presented to a private chiropractic clinic complaining of a three week history of generalized left elbow pain with no symptomatic referral to the upper extremity. The injury began immediately following a fall onto the left elbow while hiking. She could not recall the exact mechanism of her fall. The pain was described as deep and was exacerbated by weight bearing exercise and twisting motions such as opening a door. She had attempted to self-manage her symptoms with home use of a transcutaneous electrical nerve stimulation (TENS) unit, which increased her symptoms, and over the counter non-steroidal anti-inflammatory (NSAID’s) medications which had no apparent effect. She denied any history of swelling, numbness/tingling, locking sensation, or previous elbow injuries.

### Physical examination

Relevant examination findings revealed an alert and oriented, non-distressed, patient with no signs of upper extremity bracing or splinting. Active range of motion (ROM) of the left elbow was full in all directions and pain was present at end-range supination and pronation of the forearm. Palpation revealed significant tenderness slightly distal to the left radial head and along the lateral forearm, and hypertonicity of the forearm extensor muscle group was noted. To assess for lateral epicondylitis Mill’s and Cozen’s tests were performed, both provoking discomfort in the proximal forearm. The discomfort could not be reproduced at the lateral epicondyle. Valgus and varus stress testing of the elbow provoked significant discomfort distal to the lateral epicondyle, but again were not consistent with expected positive testing.

Bilateral upper extremity neurological examination was within normal limits, revealing 2+ triceps, biceps, and wrist reflexes, intact sensation to light touch of C5 to T1 dermatomes, and 5/5 motor testing of C5 to T1 myotomes. Upper limb tension test was negative for upper extremity radicular pain provocation.

### Imaging

**Figure 2:**
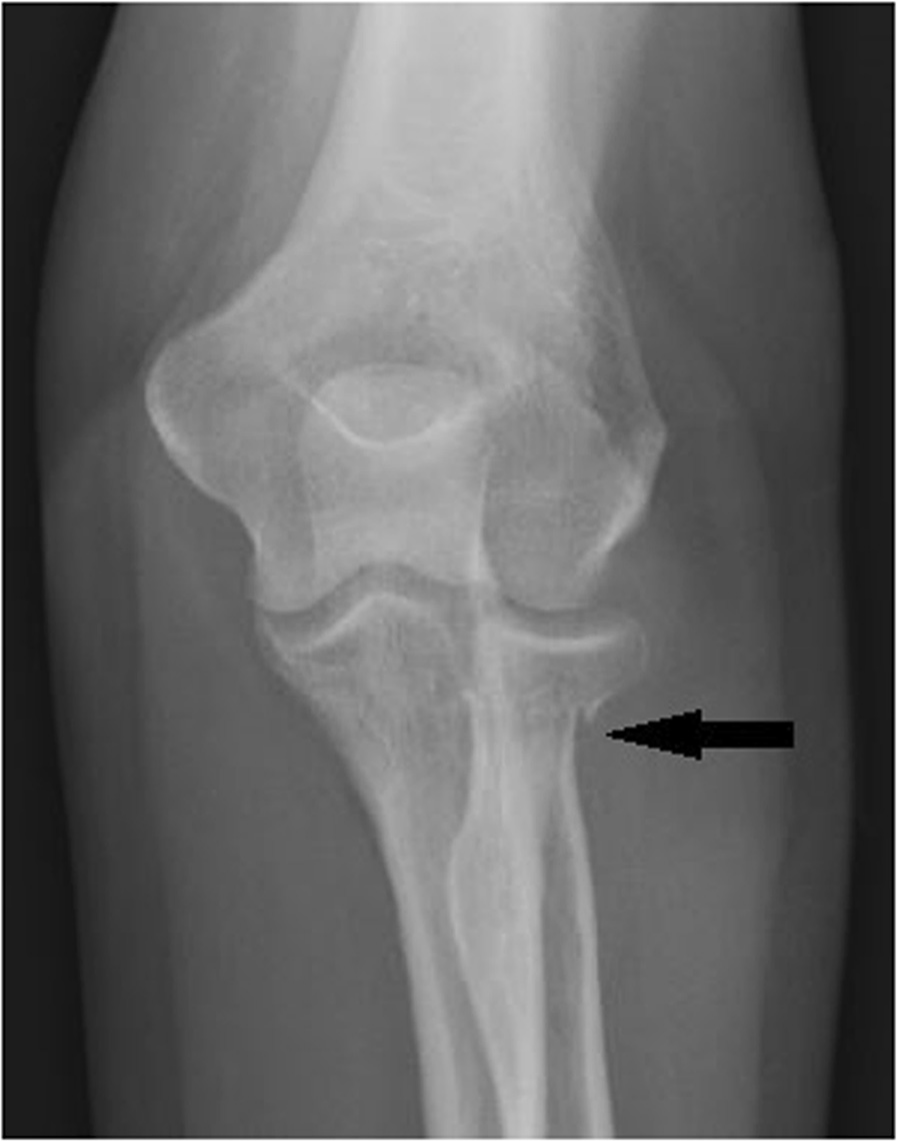
**Antero-posterior X-ray elbow view. **Note the approximately 2mm displacement of the radial head. Mason Type II Fracture.

**Figure 3:**
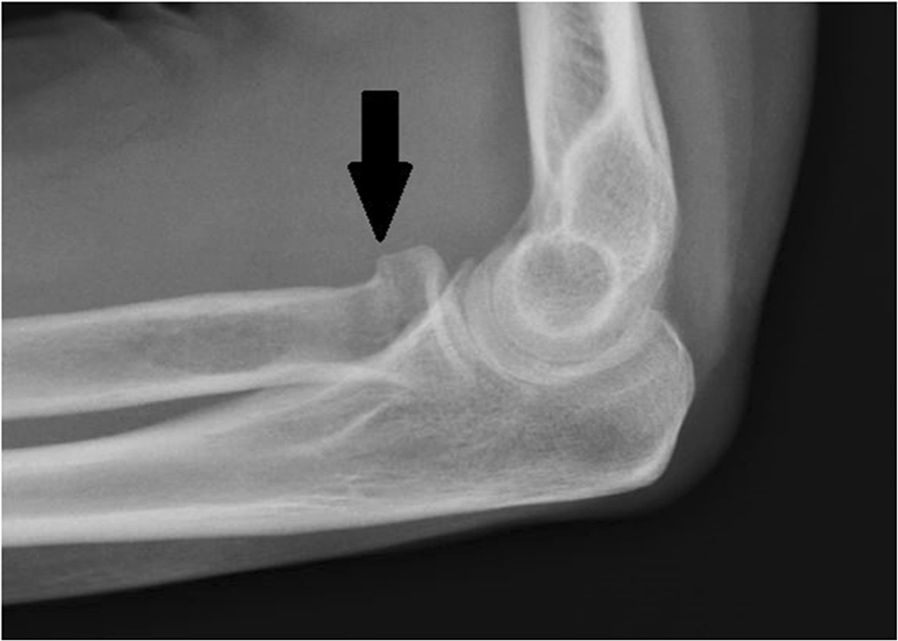
Lateral X-ray elbow view.

Due to the traumatic nature of the injury and the examination that was inconsistent for myofascial condition, she was referred to a local imaging center. Radiographs of the elbow and forearm revealed a transverse fracture through the neck of the proximal radius. There was an approximately 2mm medial displacement of the radius distal to the fracture, no evidence of dislocation, and there was suspected joint effusion (Figures 2 and 3).

### Case management

Three days post initial consultation, the patient’s X-ray results were received from the imaging center and the patient was referred to a general medical specialist for care. A clinical report and the radiologists report were forwarded to the referred physician. Verbal follow-up with the patient two-weeks later revealed that she had been referred to an orthopedic specialist by her primary care physician. The orthopedist elected not to cast the fracture due to small degree of displacement, lack of significant swelling, and range of motion within normal limits. She was restricted from performing any weight bearing exercise and was instructed to return in one month for follow-up X-ray to assess the healing of the fracture site. At four-weeks following the initial evaluation, a repeated radiographic examination demonstrated healing of the fracture site. At three month telephone follow-up the patient reported full use of her elbow with no pain in any ranges of motion.

## Discussion

The Mason classification system is used to classify radial head and neck fractures and is useful when assessing further treatment options [16]. The classification system consists of three types: Type I, non-displaced (or small marginal) radial head or neck fracture; Type II, marginal fractures with displacement including impaction, depression and angulation; and Type III, comminuted fractures involving the entire radial head [10,16,17]. Type I fractures are initially treated with 3 to 5 days of immobilization. After pain has subsided, gentle active range of motion at the elbow should begin. The immobilizing splint should be replaced by a sling as range of motion exercises begin. Follow-up radiographs are obtained to ensure that no further displacement has occurred. The patient may return to full, unrestricted activity in 3 to 4 weeks in most cases. The criterion used for return to activity is the level of comfort reported by the patient.

When Type II fractures have only a single displacement with greater than 3mm, or when the articular surface of the fracture fragment diverges more than 30 degrees, open reduction internal fixation surgery with small screws is recommended [18]. In most Mason Type II and III fractures in which displacement and/or comminution are noted on radiographs the elbow joint should be aspirated and injected with local anesthetic. This allows for careful passive range of motion examination to determine the presence or absence of mechanical blockage due to displaced fragments through the articular surface. If no mechanical blockage is identified, and if the fracture is not displaced or angulated extensively, conservative treatment is appropriate [19].

The anterior and posterior fat pads are intracapsular structures that may provide valuable information on intracapsular injuries [19]. Hemarthrosis from intra-articular fractures commonly cause the anterior fat pad to bulge further anteriorly, and the posterior fat pad will be displaced posteriorly and proximally, rendering it visible on the standard lateral x-ray projection [19]. In addition, potential injury to the supporting ligamentous structures in the elbow include sprain strain, ligament ruptures and tendon entrapments must always be considered (Table 1). The distal radioulnar joint should be examined for obvious bruising and tenderness, with special attention to the area around the medial collateral ligament and Interosseous ligament. The lateral collateral ligament stabilizes the joint against posterolateral rotational instability and is prone to injury. It is difficult to properly assess level of damage to the interosseous membrane and the resultant radioulnar instability by exam only. Diagnostic ultrasonography is a valuable tool for assessing soft tissue injuries and should be considered if ligament damage is suspected. Injuries to the median, ulnar and radial nerves as well as serious vascular claudication is possible and have been reported in Type III fractures. Therefore a thorough neurological exam of the region should be included in the patient work up.

**Table 1:** Sites of injuries with radial head fractures

**Osseous injuries**	**Soft tissue injuries**
Distal radioulnar joint fractures	Interosseous membrane disruption
Coronoid fractures	Lateral collateral ligament injuries
Carpal fractures	Medial collateral ligament injuries
Distal radioulnar joint and radial head fracture (Essex-Lopresti)	Elbow dislocation
Radial head fx, coronoid fx, elbow dislocation (terrible triad)	Vascular injuries

Conservative treatment of Type II and Type III fractures of the radial head consists of immobilization for 7 to 10 days in a posterior splint. Splinting the elbow in flexion versus extension does not affect outcome [20]. The elbow should be splinted at the angle of greatest comfort. [21] Radiographs should be repeated at appropriate intervals to ensure that further displacement or loose body formation has not occurred. Graduated mobilization may be achieved using a functional fracture brace with controlled hinges and active range of motion exercises. Several reports show excellent results using conservative treatment measures for even the most extensive comminuted radial head fractures [22,23]. The keys to success are exclusion of cases in which mechanical blockage is present and the implementation of early, controlled active range of motion [20]. After the orthopedic goals of stabilization have been achieved, the rehabilitative objectives of restoring normal ranges of motion and restoring muscle strength to the supporting musculature should be met.

The complex motion of the proximal radioulnar joint includes both pronation and supination. These movements occur in conjunction with simultaneous movement of the distal radioulnar joint [24]. Proximally, supination and pronation occur with spinning of the radial head within the fibro-osseous ring formed by the annular ligament and radial notch of the ulna [24]. During supination, the proximal surface of the articular disc remains in contact with the ulna head and at end range the palmar capsular ligament is stretched to its maximal length creating stiffness and stabilization of the joint. [24]. Whereas full pronation elongates the dorsal capsular ligament at the distal radioulnar joint and slackens the palmar capsular ligament [24]. Rehabilitation is an important part in re-establishing normal kinematics of the joint.

## Conclusion

Radial head fractures are relatively common and therefore it is important for the chiropractic physician to have knowledge of their diagnosis and treatment strategies. The clinical presentation, classification, and treatment options of radial head fractures vary depending on the amount of radial head displacement and soft tissue injury. This case study demonstrates thorough clinical examination, imaging and triage to assist in appropriate patient diagnosis and management. In this case the minimally displaced Type II Mason fracture did not require surgical intervention to return to full functionality.

### Consent

Written informed consent was obtained from the patient for publication of this case report and accompanying images. A copy of written consent is available for review by the Editor-in-Chief of this journal.

## Competing interests

The authors declare that they have no competing interests.

## Authors’ contributions

CD cared for the patient, performed the literature review, and prepared the manuscript. JG performed the literature review and assisted in preparation of the manuscript. DE assisted in manuscript preparation, developing figures and revision. All authors’ read and approved the final manuscript.
